# Evaluation of various diets for improved growth, reproductive and nutritional value of the yellow mealworm, *Tenebrio molitor* L.

**DOI:** 10.1038/s41598-025-98254-y

**Published:** 2025-05-05

**Authors:** M. A. Mahmoud, Abeer O. Abotaleb, Rasha A. Zinhoum

**Affiliations:** 1 Plant Protection Department, Faculty of Agriculture, Al-Azhar University, Assiut, Egypt; 2 Stored Product Pest Department, Plant Protection Research Institute, Agricultural Research Center, ARC, Giza, Egypt

**Keywords:** Yellow mealworm, Different diets, Weight gain, Biology, Mortality, Biological techniques, Developmental biology

## Abstract

Breeders are under growing pressure to enhance the production of farmed insects and shorten their life cycles due to the rising demand for edible insects. Feed for yellow mealworm was developed using five feed sources viz., faba bean flour, chickpea flour, wheat bran, wheat germ and Yeast, alone and in combination and resulting in 19 diet treatments. An investigation was carried out to determine the effects of diet combinations on the development, weight, and growth of *Tenebrio molitor* larvae, pupae, and adults. Results indicate that the larvae fed on diet 18 gained the most length and weight, measuring18.45 mm and 184.62 mg, respectively. The maximum length and weight, measuring 23.30 mm and 146.00 mg on diets 19 and 15 in pupae and 14.45 mm and 119.27 mg on diets 11 and 17 in adults, respectively. The shortest developmental durations were 61.00 and 6.36 days for larvae and pupae on diet 3. The shortest adult longevity was 7.00 days on diet 16. The highest fecundity rate was significance different (43.82 eggs/female) fed on diet 8 compared to the other diets. The highest percentage of adult emergence was 76.71% on diet 9. The shortest total developmental period was 88.01 days in diet 4. The maximum growth index was 2.61 in diets 6. The highest percent of protein, carbohydrate and fat contents of *T. molitor* were 49.46, 74.83 and 39.91% in larvae fed on diets 5, 10 and 3. The findings showed that wheat germ is an appropriate diet to enhance yellow mealworm production either on its own or in combination with other diets.

## Introduction

Due to the continuing growth of the world population, the shortage of food and the increase in prices of conventional protein sources such as meat and fish led an alternative source of protein, insects have been considered a viable alternative and sustainable source of nutrients for animal feed and human consumption^1–4^. Over 2000 species of insects are consumed by humans worldwide, with entomophagy still being practiced in Africa, Asia, Latin America and Australia^5,6^. In addition to having a low environmental impact and effective feed conversion rates, Insects have a high amount of protein, fat, and minerals, positioning them as a promising food source and ingredient option for enhancing the quality of various food products, in contrast to conventional primary protein sources utilized for human food and animal feed^7^.

Yellow mealworm, *Tenebrio molitor* L. (Coleoptera: Tenebrionidae) is an insect that damages stored products with a high reproduction rate, and short life cycle and as animal feed because it contains high quantities of proteins, fats, essential amino acids, vitamins and minerals^8–12^. Presently, *T. molitor* is produced for human consumption to improve the nutritional and functional properties of food products^13–15^.

Several previous studies have evaluated various diets (i.e., cereal flours and meals, non-flour, cereal commodities, legumes and various commodities of vegetative and animal origin) for the mass production of *T. molitor* significantly enhanced most biological parameters measured such as development time, fertility and percentage mortality rate and the nutritional composition of *T. molitor*^16–20^. Also, the growth and performance of insects are heavily influenced by their diet^21,22^. A species-specific diet should be designed to maximize the total larval biomass production and enhance adult reproduction performance through the development of feeds and food^23,24^. A significantly improved most biological parameters in diets with higher protein and lipid contents food^25^. A complete grain of wheat consists of bran, endosperm and germ, with the germ making up about 2.5 to 3.8% of the grain weight^26,27^. Wheat germ is the most valuable part of the wheat grain and germ is separated from the bran and starch during the milling process and is important in the food processing of high nutrition value^28,29^. It is a high source of vitamins, minerals, fiber and proteins^27,30^. Wheat germs satisfy certain nutritional requirements of several insect species^31^. Yellow mealworms have the ability it to select foods to balance their diet ratio on intake according to their nutritional needs^32^.

In this context, the main objective of this study is to rear the *T. molitor* on different substrates with wheat bran, germ and yeast, as alternative feeding substrates low cost and their effects on morphometrics (length and weight), development time, mortality rate and nutritional composition of *T. molitor*. Additionally, the effect of different diets on protein, carbohydrates and fat contents in *T. molitor* larvae was considered.

## Materials and methods

### Insect rearing

The initial population of *T. molitor* adults was obtained from the Stored Grains and Products Pests Department, Plant Protection Research Institute, Agricultural Research Center, Egypt. Before starting the experiment, the adults were reared for four generations. The colony was maintained in plastic bowls (35 × 25 × 10 cm), placed in an incubator, and kept at a constant temperature of 26 ± 1 °C and 65 ± 5% R.H., in darkness on standard wheat bran as substrate. Adults and larvae were provided with slices of fresh carrots or cabbage leaves once a week to serve as a moisture source.

### Diets preparation

Feed for yellow mealworm was developed using five feed sources distinct were purchased from the local market prior to testing viz., faba bean flour (BF), chickpea flour (CF) wheat bran (WB), wheat germ (WG) and Yeast (Y), alone and in combination and resulting in 19 diet treatments as shown in Table 1. Diets were put in clean polyethene bags and were kept at 5 °C in the refrigerator for 7 days to destroy all germs.

**Table 1:** Composition ratios of different diets combinations for rearing and feeding of *T. molitor*.

Diets	Composition ratios				
	BF	CF	WB	WG	Y
D1	1	1.25	0.125	–	0.125
D2	1.25	1	0.125	–	0.125
D3	1	–	0.125	1.25	0.125
D4	–	1	0.125	1.25	0.125
D5	1.25	–	0.125	1	0.125
D6	–	1.25	0.125	1	0.125
D7	1	–	1.4	–	0.4
D8	–	1	1.4	–	0.4
D9	–	–	1.4	1	0.4
D10	1	–	0.6	–	0.25
D11	–	1	0.6	–	0.25
D12	–	–	0.6	1	0.25
D13	1	–	0.19	–	0.063
D14	–	1	0.19	–	0.063
D15	–	–	0.19	1	0.063
D16	1	–	–	–	0.05
D17	–	1	–	–	0.05
D18	–	–	–	1	0.05
D19 (Control)	–	–	1	–	–

### Experimental design

Each diet (500 g) was placed into clean plastic boxes (35 × 25 × 10 cm). 30 mixed-sex adults of yellow mealworm were placed into each rearing box to allow for mating and oviposition, and slices of fresh carrots or cabbage leaves were provided once a week. After 15 days, the adults were removed, and their larvae were collected using a sterile standard sieve for all rearing boxes. For all bioassays, plastic boxes remained until pupation at 26 ± 1 °C and 65 ± 5% R.H., in an incubator. Each diet box was replicated three times making 57 rearing boxes. The number of yellow mealworms produced, and the fecundity (number of young produced per female) were then assessed for each treatment. The fecundity (F) was calculated using the formula; F = NI / NF, where NI is the number of individuals produced and NF is the number of females. All the larvae produced in each treatment were weighed and measured for length at the beginning of the experiment, and their initial weight and length were recorded. The mortality%, duration and pupation% were determined for larvae, pupa and adults. After pupation, the emerged adults were counted for all diets.

### Nutrient composition analysis

At the end of the larvae instar for each diet, the 10 larvae of each box were separated from the diet and kept without food for 12 h then were placed in the deep freezer to determine the contents of protein, carbohydrate and fat. The protein was determined according to Gornall et al.^33^. Total carbohydrates were estimated in an acid extract of insects by the phenol–sulphuric acid reaction (Dubios et al.^34^). Total carbohydrates were extracted and prepared for assay according to Crompton & Birt^35^. Total lipids were estimated by the method described by Zöllner and Kirsch^36^.

### Statistical analysis

Prior to analysis, Shapiro–Wilk’s test was done for the assumption of normality in which the test was insignificant. Collected data were subjected to the Analysis of Variance (ANOVA) using Statistical Analysis System (SAS). The mean differences were separated using Tukey–Kramer HSD test, and showed as means ± SE.

## Results

### Influence of various diets on the growth and development of *T. molitor* larvae

Data in Table 2 demonstrated the average length, weight, development period and mortality of larvae of *T. molitor*. Results indicated that all these parameters were significantly (P = 0.05) affected by tested diets under laboratory conditions. The maximum body length gain was recorded at 18.45 mm in larvae fed on diet 18 followed by diet 14 with a length gain of 16.90 mm, while the minimum length gain was 6.65 mm in larvae fed on diet 5. The minimum weight gain was recorded at 72.07 mg for larvae fed on diet 19 followed by diet 3 with a weight of 74.61 mg, while the maximum weight gain was 185.54 and 184.62 mg for larvae fed on diet 15 and 18, respectively. Statistical analysis showed the various diets has a significant effect on the length gain of larvae (F = 25.70; df = 379; P < 0.001) and larvae weight gain (F = 5.67; df = 379; P < 0.001); Table 2. *T. molitor* larvae fed diet 19 had the longest duration (100.33 days), followed by those fed diet 10, which had a lifespan of 95.66 days. In contrast, larvae that were fed diets 3 and 4 on the other hand lived for 61.00 and 63.00 days, respectively. The highest percent of pupation of *T. molitor* observed in diet 1 was 95.82%, while the minimum percent of pupation was 51.71% in diet 8. There was a significant various diets impact on duration of larvae (F = 44.80; df = 56; P < 0.001) and pupation (F = 7.16; df = 56; P < 0.001); Table 2. The lowest percent mortality was 2.80, 3.21 and 3.71% fed on diets 3, 5 and 18 respectively; while the highest percent mortality was 48.90% for larvae fed on diet 13. Statistical parameters of larvae mortality (F = 18.40; df = 56; P < 0.001); Table 2.

**Table 2:** Nutritional impact of various diets on the developmental aspects, pupation and mortality rates of *T. molitor* larvae.

Diets	Mean ± SE				
	Length (mm)	Weight (mg)	Duration (days)	Pupation (%)	Mortality (%)
D1	11.40 ± 0.55^def^	84.86 ± 3.44^cd^	83.00 ± 1.52^def^	95.82 ± 1.54^a^	4.37 ± 1.14^cd^
D2	10.05 ± 0.76^ef^	145.81 ± 16.82^abcd^	86.00 ± 1.73^cde^	94.33 ± 1.75^a^	7.25 ± 0.57^cd^
D3	9.75 ± 0.59^fg^	74.61 ± 5.68^d^	61.00 ± 1.00^i^	75.33 ± 6.44^abc^	2.80 ± 0.65^d^
D4	8.45 ± 0.72^fg^	80.42 ± 4.25^d^	63.00 ± 1.00^i^	84.50 ± 2.12^a^	7.21 ± 0.33^cd^
D5	6.65 ± 0.71^g^	88.06 ± 6.59^cd^	73.33 ± 1.76^gh^	88.10 ± 0.95^a^	3.21 ± 0.75^d^
D6	9.35 ± 0.47^fg^	105.85 ± 15.36^bcd^	71.00 ± 0.57^h^	78.48 ± 1.09^ab^	4.34 ± 1.10^cd^
D7	14.65 ± 0.58^bc^	180.44 ± 47.68^ab^	83.00 ± 1.52^def^	82.52 ± 4.23^a^	8.81 ± 1.22^bcd^
D8	14.05 ± 0.77^bcd^	117.63 ± 13.03^abcd^	83.00 ± 0.57^def^	51.71 ± 2.1^c^	9.06 ± 0.92^bcd^
D9	14.85 ± 0.48^bc^	123.71 ± 3.38^abcd^	76.66 ± 1.76^fgh^	91.47 ± 3.52^a^	10.94 ± 1.13^bcd^
D10	13.15 ± 0.81^cde^	137.75 ± 3.50^abcd^	95.66 ± 1.76^ab^	91.04 ± 7.31^a^	4.56 ± 0.85^cd^
D11	16.20 ± 0.44^abc^	138.07 ± 5.68^abcd^	85.00 ± 0.57^de^	93.87 ± 2.39^a^	10.54 ± 1.51^bcd^
D12	14.75 ± 0.44^bc^	169.16 ± 10.06^ab^	76.66 ± 2.40^fgh^	82.07 ± 3.42^a^	10.38 ± 0.54^bcd^
D13	14.30 ± 0.51^bcd^	160.95 ± 32.73^abc^	80.66 ± 2.40^efg^	56.00 ± 12.50^bc^	48.90 ± 8.18^a^
D14	16.90 ± 0.44^ab^	138.00 ± 8.88^abcd^	84.33 ± 0.88^def^	90.50 ± 1.01^a^	16.44 ± 2.50^bc^
D15	15.40 ± 0.53^abc^	185.54 ± 8.02^a^	93.00 ± 2.51^abc^	90.32 ± 2.89^a^	6.67 ± 1.42^cd^
D16	15.30 ± 0.57^abc^	129.81 ± 6.75^abcd^	90.33 ± 0.88^bcd^	85.54 ± 5.15^a^	20.43 ± 2.79^b^
D17	16.60 ± 0.89^ab^	119.91 ± 9.42^abcd^	82.66 ± 1.20^def^	80.76 ± 3.57^a^	15.53 ± 3.55^bcd^
D18	18.45 ± 0.75^a^	184.62 ± 7.14^a^	81.33 ± 0.88^ef^	93.30 ± 1.00^a^	3.71 ± 0.41^cd^
D19	11.35 ± 0.74^def^	72.07 ± 6.07^d^	100.33 ± 0.88^a^	79.50 ± 4.30^ab^	12.15 ± 2.21^bcd^

Means, in the same column, followed by the same letter are not significantly different using the Tukey–Kramer HSD test at P = 0.05.

### Influence of various diets on the growth and development of *T. molitor* pupae

Results presented in Table 3 showed that all pupae parameters were significantly affected (P = 0.05) by the tested diets. However, the minimum pupae length was 14.25 mm in diet 3 and 14 respectively, while the maximum pupae length was 23.30 mm in diet 19. The maximum pupae weight observed was 146.00 mg in diet 15, whereas the minimum pupae weight was 93.53 mg in diet 2 compared to the other diets. There was a significant various diets impact on pupae length (F = 39.00; df = 379; P < 0.001) and pupae weight (F = 5.44; df = 379; P < 0.001); Table 3. The longer pupae duration of *T. molitor* was 9.25 days in diet 19 which was closely followed by diets 7 and 10 of 9.15 days, while the shorter duration was 6.36 days in diet 3. The highest percent of pupae mortality was 40.78% in diet 13, while the lowest percent of pupae mortality was 2.90% in diet 3, followed by diet 10 was 3.57%. Statistical analysis showed the various diets has a significant effect on pupae duration (F = 5.47; df = 56; P < 0.001) and pupae mortality (F = 13.50; df = 56; P < 0.001); Table 3.

**Table 3:** Nutritional impact of various diets on the developmental aspects and mortality rates of *T. molitor* pupae.

Diets	Mean ± SE			
	Length (mm)	Weight (mg)	Duration (days)	Mortality (%)
D1	14.85 ± 0.41^d^	114.50 ± 5.32^bcdef^	6.98 ± 0.01^bcd^	5.84 ± 1.93^fg^
D2	14.31 ± 0.24^d^	93.53 ± 9.10^f^	8.75 ± 0.62^ab^	6.06 ± 1.18^fg^
D3	14.25 ± 0.27^d^	135.19 ± 6.36^abc^	6.36 ± 0.36^d^	2.90 ± 0.5^g^
D4	17.25 ± 0.56^c^	121.34 ± 3.48^abcde^	8.01 ± 0.59^abcd^	9.60 ± 0.63^efg^
D5	15.50 ± 0.43^cd^	125.79 ± 3.33^abcde^	8.01 ± 0.01^abcd^	3.61 ± 0.63^fg^
D6	15.30 ± 0.26^cd^	132.70 ± 3.72^abcd^	6.86 ± 0.18^cd^	5.38 ± 0.64^fg^
D7	14.65 ± 0.29^d^	114.68 ± 3.60^bcdef^	9.15 ± 0.15^a^	18.41 ± 1.42^bcdef^
D8	15.00 ± 0.48^d^	104.52 ± 5.36^ef^	8.13 ± 0.59^abcd^	6.94 ± 0.69^fg^
D9	15.40 ± 0.31^cd^	111.66 ± 7.23^bcdef^	8.46 ± 0.29^abc^	10.61 ± 0.86^defg^
D10	14.40 ± 0.31^d^	128.88 ± 5.26^abcde^	9.15 ± 0.45^a^	3.57 ± 0.16^fg^
D11	16.10 ± 0.20^cd^	127.34 ± 2.94^abcde^	8.71 ± 0.28^abc^	12.18 ± 0.5^cdefg^
D12	21.10 ± 0.66^b^	124.06 ± 7.99^abcde^	7.85 ± 0.14^abcd^	27.12 ± 1.19^ab^
D13	16.05 ± 0.41^cd^	106.28 ± 4.04^def^	8.01 ± 0.01^abcd^	40.78 ± 7.94^a^
D14	14.25 ± 0.41^d^	109.26 ± 2.95^cdef^	7.81 ± 0.15^abcd^	10.65 ± 1.84^defg^
D15	21.45 ± 0.65^ab^	146.00 ± 5.98^a^	7.70 ± 0.15^abcd^	18.40 ± 1.85^bcdef^
D16	16.05 ± 0.49^cd^	125.59 ± 5.49^abcde^	8.36 ± 0.31^abc^	22.34 ± 2.25^bcde^
D17	14.90 ± 0.33^d^	125.37 ± 4.8^abcde^	9.08 ± 0.50^a^	26.10 ± 2.90^abc^
D18	14.95 ± 0.55^d^	137.81 ± 5.6^ab^	7.46 ± 0.29^abcd^	25.25 ± 6.28^bcd^
D19	23.30 ± 0.38^a^	125.96 ± 7.17^abcde^	9.25 ± 0.37^a^	15.69 ± 3.84^bcdefg^

Means, in the same column, followed by the same letter are not significantly different using the Tukey–Kramer HSD test at P = 0.05.

### Influence of various diets on *T. molitor* adult’s parameters

The obtained data in Table 4 represent the adult parameters of *T. molitor* fed on different diets under laboratory conditions. Statistical analysis shows that there were significant differences in all among tested diets (p = 0.05). Results indicate that the maximum adult length was recorded at 14.45 mm in diet 11 followed by diet 15 (14.35 mm), while the minimum adult length was 12.55 and 12.60 in diets 6 and 12 respectively. The maximum adult weight (120.97 mg) was recorded in diet 15 which was closely followed by diet 3 (120.49 mg), whereas the minimum adult weight (91.31 mg) was recorded in diet 13 compared to the other diets. The longer adult longevity of adults was at observed 26.33 days in diet 5 followed by diet 3 and 18 was 25.66 days, while the shorter adult longevity was 7.00 days in diet 16. The highest fecundity rate (number of laid eggs/female) was recorded at 43.82 eggs/female in females fed on diet 8, while the lowest fecundity rate was 7.88 eggs/female in females fed on diet 13. Statistical analysis shows that there were significant differences in all among tested diets on adult length (F = 2.52; df = 379; P < 0.001), adult weight (F = 5.48; df = 379; P < 0.001); adult longevity (F = 11.00; df = 56; P < 0.001); fecundity rate (F = 36.40; df = 56; P < 0.001); Table 4.

**Table 4:** Nutritional impact of various diets on the developmental aspects and fecundity of *T. molitor* adults.

Diets	Mean ± SE			
	Length (mm)	Weight (mg)	Adult longevity (days)	Fecundity
D1	13.25 ± 0.20^abc^	100.59 ± 2.56^abcd^	9.33 ± 1.20^def^	21.20 ± 0.65^cdef^
D2	13.25 ± 0.28^abc^	105.18 ± 4.73^abcd^	19.00 ± 2.08^abcd^	25.28 ± 2.07^bcd^
D3	12.95 ± 0.27^abc^	120.49 ± 5.80^a^	25.66 ± 2.33^ab^	21.42 ± 0.05^cdef^
D4	13.40 ± 0.49^abc^	116.41 ± 6.13^abc^	17.00 ± 1.73^abcde^	9.28 ± 0.45^hi^
D5	13.35 ± 0.20^abc^	118.15 ± 3.22^ab^	26.33 ± 2.40^a^	15.88 ± 0.24^fgh^
D6	12.55 ± 0.18^c^	94.30 ± 5.47^cd^	16.00 ± 0.57^bcdef^	30.11 ± 1.85^b^
D7	12.90 ± 0.25^abc^	94.42 ± 2.12^cd^	9.33 ± 1.20^def^	14.31 ± 0.79^fghi^
D8	13.20 ± 0.28^abc^	92.65 ± 3.69^d^	9.66 ± 1.45^def^	43.82 ± 1.92^a^
D9	13.20 ± 0.29^abc^	109.88 ± 6.19^abcd^	19.00 ± 2.08^abcd^	25.08 ± 1.09^bcd^
D10	13.25 ± 0.37^abc^	93.02 ± 4.4^d^	13.66 ± 1.33^cdef^	24.35 ± 2.74^bcde^
D11	14.45 ± 0.43^a^	105.06 ± 3.59^abcd^	22.66 ± 1.45^abc^	25.22 ± 1.55^bcd^
D12	12.60 ± 0.31^c^	108.37 ± 5.49^abcd^	12.33 ± 1.45^def^	29.42 ± 1.10^b^
D13	13.65 ± 0.56^abc^	91.31 ± 5.09^d^	14.33 ± 3.48^cdef^	7.88 ± 1.06^i^
D14	12.75 ± 0.29^bc^	99.40 ± 3.67^abcd^	15.00 ± 0.00^cdef^	27.04 ± 2.73^bc^
D15	14.35 ± 0.41^ab^	120.97 ± 2.34^a^	16.66 ± 2.02^abcdef^	12.46 ± 0.43^ghi^
D16	13.95 ± 0.19^abc^	97.57 ± 3.74^bcd^	7.00 ± 1.52^f^	14.66 ± 1.01^fghi^
D17	13.75 ± 0.32^abc^	119.27 ± 6.32^ab^	8.66 ± 0.88^ef^	11.66 ± 1.20^ghi^
D18	13.60 ± 0.36^abc^	114.09 ± 4.32^abcd^	25.66 ± 2.18^ab^	17.91 ± 0.40^defg^
D19	12.85 ± 0.31^abc^	92.38 ± 5.31^d^	19.0 ± 2.08^abcd^	16.80 ± 1.87^efgh^

Means, in the same column, followed by the same letter are not significantly different using the Tukey–Kramer HSD test at P = 0.05.

The obtained data in Table 5 represent the adult parameters of *T. molitor* fed on different diets under laboratory conditions. Results indicate that the higher numbers of adult were emerged in diet 6 was 289.00 adults, while the minimum number of adults were emerged was 13.00 adults in diet13. The minimum percent of adult emergence was recorded at 9.58% in diet 13, while the highest percent of adult emergence in diet 9 was 76.71%. The longest total developmental period of *T. molitor* was 128.58 days in diet 19 and while, the shortest total developmental period was 88.01 days in diet 4. The maximum growth index was 2.61 in diets 6, while, the minimum growth index was 0.89 in diet 13. Statistical analysis shows that there were significant differences of numbers of adult in all among tested diets (F = 35.40; df = 56; P < 0.001); percent of adult emergence (F = 21.80; df = 56; P < 0.001); total developmental period of *T. molitor* (F = 21.90; df = 56; P < 0.001); growth index (F = 22.10; df = 56; P < 0.001); Table 5.

**Table 5:** Nutritional impact of various diets on the number of adult, total development period and growth index of *T. molitor* adults.

Diets	Mean ± SE			
	Number of adult emerged	Adult emergence (%)	Development period (days)	Growth index
D1	235.00 ± 17.59^ab^	73.96 ± 5.45^ab^	99.31 ± 2.59^efg^	2.38 ± 0.08^abc^
D2	255.66 ± 22.28^a^	67.43 ± 2.66^abc^	113.75 ± 1.84^bcd^	2.11 ± 0.01^bcde^
D3	86.66 ± 10.94^defg^	26.98 ± 3.46^hi^	93.03 ± 3.03^fg^	2.07 ± 0.06^bcdef^
D4	95.00 ± 2.519^defg^	68.47 ± 1.39^abc^	88.01 ± 2.09^g^	2.24 ± 0.06^abcd^
D5	74.66 ± 9.53^defg^	31.33 ± 3.93^fgh^	107.68 ± 3.51^bcde^	1.73 ± 0.07^efg^
D6	289.00 ± 17.91^a^	63.98 ± 0.62^abcd^	93.86 ± 1.01^fg^	2.61 ± 0.01^a^
D7	116.00 ± 16.52^def^	53.64 ± 5.05^bcde^	101.48 ± 0.48^ef^	2.02 ± 0.06^cdefg^
D8	282.66 ± 8.96^a^	43.06 ± 0.83^defgh^	100.80 ± 2.44^ef^	2.43 ± 0.06^abc^
D9	286.66 ± 15.91^a^	76.71 ± 6.84^a^	104.13 ± 4.04^def^	2.36 ± 0.11^abc^
D10	153.00 ± 14.24^bcd^	42.67 ± 5.06^efgh^	118.48 ± 2.45^ab^	1.84 ± 0.05^defg^
D11	219.00 ± 12.13^abc^	57.99 ± 1.85^abcde^	116.38 ± 1.69^bc^	2.01 ± 0.05^cdefg^
D12	263.66 ± 34.42^a^	59.53 ± 6.54^abcde^	96.85 ± 1.14^efg^	2.49 ± 0.08^ab^
D13	13.00 ± 8.51^g^	9.58 ± 5.21^i^	103.01 ± 2.65^def^	0.89 ± 0.26^h^
D14	240.33 ± 20.04^a^	59.62 ± 2.92^abcde^	107.15 ± 0.91^bcde^	2.21 ± 0.01^abcd^
D15	106.66 ± 8.22^def^	56.88 ± 2.38^abcde^	117.36 ± 0.75^abc^	1.72 ± 0.03^efg^
D16	62.33 ± 6.34^efg^	28.97 ± 4.64^ghi^	105.70 ± 2.35^cde^	1.69 ± 0.07^efg^
D17	42.00 ± 6.25^fg^	24.11 ± 3.42^hi^	100.41 ± 0.30^ef^	1.60 ± 0.06^g^
D18	139.66 ± 3.76^cde^	51.97 ± 0.32^cdef^	114.46 ± 1.46^bcd^	1.87 ± 0.02^defg^
D19	124.60 ± 20.10^de^	48.90 ± 3.48^cdefg^	128.58 ± 2.34^a^	1.62 ± 0.08^fg^

Means, in the same column, followed by the same letter are not significantly different using the Tukey–Kramer HSD test at P = 0.05.

### Impact of various diets on protein, carbohydrates and fat composition in *T. molitor* larvae

Data presented in Table 6 showed the percent of protein, carbohydrate and fat contents of *T. molitor* larvae. The results showed that there was a significant difference between the composition of the larvae were greatly influenced by tested diets, the highest percent crude protein content was recorded at 49.46% in larvae fed on diet 5, and while it was lower at 2.62% in larvae fed on diet 17, while protein content in larvae fed on other diets (diet 3 to diet 1) ranged from 49.26 to 4.60%. The highest percent carbohydrate content was recorded 74.83% in larvae fed on diet 10, while it was lower 20.53% in larvae fed on diet 13. The lowest percent crude fat content was 10.29% in larvae fed on diet 13, while it was higher at 39.91% in larvae fed on diet 3. Fat content in larvae fed on other diets (diet 9 to diet 6) ranged from 37.09 to 13.49%. There was a significant various diets impact on composition in *T. molitor* larvae of protein (F = 349; df = 56; P < 0.001); carbohydrates (F = 37.70; df = 56; P < 0.001); fat (F = 9.01; df = 56; P < 0.001); Table 6.

**Table 6:** Impact of various diets on protein, carbohydrates and fat composition in *T. molitor* larvae.

Diets	Mean ± SE		
	Protein (%)	Carbohydrates (%)	Fat (%)
D1	4.60 ± 0.77^hi^	30.13 ± 2.18^hijk^	20.42 ± 2.54^bcde^
D2	9.74 ± 1.03^fg^	32.47 ± 1.21^ghijk^	17.60 ± 2.19^cde^
D3	49.26 ± 0.87^a^	40.75 ± 1.06^efgh^	39.91 ± 4.96^a^
D4	21.55 ± 0.55^cd^	49.49 ± 1.30^cde^	15.89 ± 1.97^de^
D5	49.46 ± 0.88^a^	34.02 ± 3.32^fghij^	13.61 ± 1.69^de^
D6	23.37 ± 0.69^c^	35.34 ± 1.17^fghi^	13.49 ± 1.67^de^
D7	12.70 ± 0.85^ef^	42.43 ± 1.05^efg^	17.90 ± 2.22^cde^
D8	7.23 ± 0.67^gh^	44.95 ± 2.28^def^	17.72 ± 2.20^cde^
D9	13.24 ± 0.52^ef^	48.44 ± 1.59^cde^	37.09 ± 4.61^a^
D10	6.74 ± 0.84^gh^	74.83 ± 6.65^a^	19.61 ± 2.44^cde^
D11	6.89 ± 0.68^gh^	45.61 ± 1.09^cdef^	35.99 ± 4.48^ab^
D12	13.81 ± 0.70^e^	55.58 ± 1.91^bcd^	14.44 ± 1.79^de^
D13	24.15 ± 0.56^c^	20.53 ± 1.06^k^	10.29 ± 1.28^e^
D14	6.70 ± 0.58^gh^	22.99 ± 1.12^jk^	20.11 ± 2.50^cde^
D15	15.43 ± 0.68^e^	57.55 ± 2.71^bc^	27.80 ± 3.46^abcd^
D16	12.78 ± 0.69^ef^	50.94 ± 2.47^bcde^	20.71 ± 2.57^bcde^
D17	2.62 ± 0.43^i^	62.85 ± 1.41^ab^	31.59 ± 3.93^abc^
D18	32.23 ± 0.71^b^	24.78 ± 1.39^ijk^	27.85 ± 3.46^abcd^
D19	19.49 ± 0.79^d^	39.86 ± 1.18^efgh^	15.17 ± 1.88^de^

Means, in the same column, followed by the same letter are not significantly different using the Tukey–Kramer HSD test at P = 0.05.

## Discussion

Results indicate that the larvae fed on diet 18 gained the most length and weight, measuring18.45 mm and 184.62 mg, respectively. Shortest duration larva was 61.00 days on diet 3, as reported by these researchers; Morales-Ramos et al.^37^ observed that the length and weight of the larvae were significantly impacted by meals high in protein. Park et al.^38^ found a significantly higher survival rate of *T. molitor* fed on wheat bran compared to other diets. Kim et al.^39^ reported that larvae fed on wheat bran had the shortest developmental time compared to those on other tested diets. Oonincx et al.^16^ reported that the development time of larvae reared on low-protein diets is longer than those reared on high-protein diets. Zhang et al.^40^ observed significant differences in the weight of the larvae on diets consisting of wheat bran, corn stover, soybean meal and distiller grain. The lowest weight gain was recorded in corn stover, whereas wheat bran had the highest weight gain. Rumbos et al.^18^ mentioned that the total weight of yellow mealworm larvae produced by the different legume flours, which has protein contents ranging from 22.9 to 42.4% was found to be low. While the total larval weight produced by the different amylaceous such as wheat bran, zea flour, durum wheat flour, and white flour with protein contents between 11.1 and 14.2% was high. Naser El Deen and Lamaj^41^ found that the total mean larval weight was significantly higher for larvae reared on diets A (3.1 mg) composed of wheat bran, brewer’s yeast and G (3.2 mg) composed of wheat bran, brewer’s yeast and spent grain. Amin and Usman^42^ reported that *T. molitor* larvae fed upon standard diet-13 (wheat bran 95% + yeast 5%) and diet-5 (Rice straw 40% + wheat bran 55% + Yeast 5%) appeared to be the most effective diet have maximum weight gain, length and quick development larval as compare to other diets. Saadiya and Defrawy^19^ found the shortest length larval in corn stover and the longest in wheat bran. Larvae fed on wheat bran had the highest gain weight, while the lowest larvae weight was found in diet corn stover. Also, the highest survival rate of yellow mealworm in diets mixed with wheat bran. Riaz et al.^43^ mentioned that the average larval weight of 0.22 g fed with 70% WB and 30% brewer’s spent grain, compared to the heaviest larva of *T. molitor* fed with 100% wheat bran weighed 0.07 g. Shah et al.^44^ mentioned that larval mortality was significantly higher on a diet with porridge alone, compared to a control diet.

Our results the maximum pupae weight observed 146.00 mg in diet 15, while the lowest percent of pupae mortality was 2.90% in diet 3. The data of this work is in the same line as Morales-Ramos et al.^37^ mentioned that pupal weight in *T. molitor* is correlated with the number of stadia it takes to complete larval development and the number of stadia is correlated with development time and pupal weight ranged from 0.13 to 0.15 g. Park et al.^38^ and Kim et al.^45,46^ observed that the highest weight in *T. molitor* pupal was reared on wheat bran compared to other diets. Amin and Usman^42^ found that the survival rate of the *T. molitor* pupae was greatly affected by the diet composition. The highest survival rate of *T. molitor* pupae was (100%) in diets D-2 (Rice straw 40% + Corn straw 40% + Wheat bran 15% + Yeast 5%), D-7 (Rice straw 60% + Wheat bran 35% + Yeast 5%) and D-8 (Corn straw 60% + Wheat bran 35% + Yeast 5%). Riaz et al.^43^ reported that maximum pupal weight was recorded for pupae fed with wheat bran amounting to 0.11 g. Shah et al.^44^ found that pupae of *T. molitor* were greater on diets containing wheat bran alone and wheat bran in combination with maize and porridge.

Our results and several previous studies have shown that *T. molitor* growth, development and survival is directly influenced by feed composition. Similarly, Morales-Ramos et al.^47^ mentioned that adult size in *T. molitor* is significantly affected by nutrition. Oonincx et al.^16^ and van Broekhoven et al.^48^ studied survival rate of yellow mealworm on numerous diets and indicated that adding to wheat bran improves survival. Studies conducted by van Broekhoven et al.^48^ found the shortest developmental time of yellow mealworm on diets containing rich protein as compared to the protein-low diets. Kim et al.^45^ found that in wheat bran supplement diets 95% survival rate of yellow mealworm. Ortiz et al.^49^ concluded that *T. molitor* obtained all the required growth nutrients and development from wheat bran. Mlˇcek et al.^50^ reported that the basic feed for *T. molitor* is wheat bran, due to the observed low larval mortality of 45% over 21 weeks. and the higher speed of *T. molitor* development. Naser El Deen et al.^51^ recorded that rearing yellow mealworm on wheat bran supplemented with yeast has been shown to improve adult survival. Saadiya & Defrawy^19^ studied survival rate of yellow mealworm on numerous diets and reported highest survival rate in diets mixed with wheat bran. Bordiean et al.^52^ reported all diets contained wheat bran (except rye bran 100), even a 30% share of another byproduct could significantly influence the growth and development of the yellow mealworm. Park et al.^38^, Morales-Ramos et al.^47^ and Nukmal et al.^53^ revealed that the fecundity of *T. molitor* is greatly dependent on both pupal adult weight. Also, more eggs were deposited by the females fed on different diets supplemented with wheat bran. Kim et al.^54^ reported that the pupation rate was above 90% for larvae grown on all ratios and brewer´s spent grain as a single substrate gave the highest pupation rate of 100%. Rumbos et al.^18^ mentioned that adult reproduction was favored by most amylaceous substrates tested, which contrasted with the tested legumes on which fewer offspring were produced. Morales-Ramos et al.^55^ found that soybeans led to a pupation rate of only 2% and no emerging beetles. Amin & Usman^42^ indicated that the total number of eggs deposited by a female in diets D-2, D-3, D-7 and D-10 was non-significant in comparison to the overall fecundity of females in other diets. The maximum number of eggs per female per day (9.71) was observed in diet D-13.

Our results the highest percent of protein, carbohydrate and fat contents of *T. molitor* were 49.46, 74.83 and 39.91% in larvae fed on diets 5, 10 and 3. Rumpold and Schlüter^56^ reported high crude protein values of *T. molitor* larvae of 43.1% or 43.7%. Also, van Broekhoven et al*.*^48^ found that protein content ranges from 18.9 to 27.6%. Zhang et al.^40^ observed lower protein efficiency ratio when they fed soy bean meal to yellow mealworm larvae with high protein (43.18%) and amino acid (2.708 mg/g protein) content. Mancini et al.^57^ indicated that the crude protein content of *T. molitor* larvae was affected by the diet, as the low content of crude protein in cookies (6.55%) negatively influenced the growth and nutritional composition of the larvae with the result of the lowest weight (87 mg per larva) and larval protein content (37.31% dry matter). Canavoso et al.^58^ mentioned that protein is utilized most efficiently for tissue growth at the highest possible ratio, while carbohydrates and lipids are utilized for energy, as this produces the most rapid increase in protein body mass. Morales-Ramos et al*.*^25^ mentioned that diets with higher protein and lipid contents significantly improved most biological parameters determined, in comparison to diets containing a high carbohydrate content. Raheem et al.^59^ reported that the fat content of *T. molitor* larvae ranged from 39.1% to 40.5% depending on the diet. Melis et al.^60^ found amounts of choline-derived compounds such as betaine, DMA and sarcosine in brewer’s spent grain than in wheat bran. This accumulation can be in turn related to the observed reduction of fat storage in the form of triacylglycerols (TAGs) in brewer’s spent grain. Adámková et al.^61^ found that the fat content ranged from 16.6 to 29.5% and the amino acid content of yellow mealworm larvae after feeding a mixture of wheat bran and lentil flour resulted in higher amino acid content compared with the control group which was fed pure wheat bran. Fondevila and Fondevila^62^ where substrate mixtures based on barley straw and wheat grain with increasing contents of soybean meal were fed. This also led to greater larval protein content with higher dietary protein content. Kröncke et al.^63^ mentioned that the supplementation of pea protein resulted in higher dietary fat content and lower dietary carbohydrate content, which can reduce larval growth. A weak relationship between the protein level in the larva and those in the substrates was noted by Rumbos et al.^18^. Bordiean et al.^52^ noted that while the protein level of yellow mealworm larvae diets was almost the same, there were substantial differences in the protein composition of the larvae themselves.

In conclusion, the present study contribute to the development of diets and dietary formulations showed good results in the development and growth of *T. molitor* and also, impacted of various diets on composition of *T. molitor* larvae. Wheat germ is an appropriate diet its own or in combination with other diets to enhance yellow mealworm production on a large scale for use in food purposes for humans and animals, due to its high content of protein and fat, and it is an easy and cheap source for large-scale protein production.

## Data Availability

The datasets used and/or analyzed during the current study are available from the corresponding author on reasonable request.
